# Preparing students for diverse careers in the poultry industry

**DOI:** 10.1016/j.psj.2024.104485

**Published:** 2024-11-13

**Authors:** Elizabeth L. Karcher, J. Marcos Fernandez, Christine Z. Alvarado, Nada Tamim, Drew Benson

**Affiliations:** a Purdue University, West Lafayette, IN, USA; b Prosur Inc, Naperville, IL, USA; c University of Georgia, Georgia, USA

**Keywords:** AI technologies, Careers, Undergraduate, Workforce development

## Abstract

Careers in the poultry industry are ever evolving to address global challenges and include collaborations with others from diverse backgrounds and cultures from around the world. Employers are seeking college graduates who are prepared to use novel approaches to address local and global industry challenges. A primary goal of undergraduate animal and poultry science programs should be to focus on courses and opportunities for students to engage in activities that promote workforce and essential skill development. A symposium at the 2024 Poultry Science Association annual meeting included a unique perspective from academic and industry professionals on the essential skills needed by graduates to be most successful in the industry. This paper is a summary of the symposium's presentations concerning workforce development.

## Session 1: Employment opportunities remain strong for college graduates in the food, agriculture, renewable natural resources, and environment disciplines

J. Marcos Fernandez

According to the most recent USDA National Institute of Food and Agriculture report on employment opportunities for college graduates in the food, agriculture, renewable natural resources and environmental (FARNRE) disciplines (Fernandez et al., 2020), there will be approximately 59,400 employment opportunities annually between 2020 and 2025, representing a 2.6 % improvement over the previous five-year period (Goecker et al., 2015). Annual employment opportunities were determined using Bureau of Labor Statistics Projections between 2018 and 2028 across 78 employment sectors identified by the research team, and the results showed 24,700 positions in business and management, 18,400 positions in science and engineering, 8,400 openings in education, communication and government positions, and 7,900 in food, biomaterials and production positions, accounting for 42, 31, 14 and 13 percent of the annual openings, respectively (Fernandez et al., 2020).

The supply of available graduates (includes baccalaureate, masters, doctorate, and professional veterinary medicine degrees) entering the workforce from FARNRE academic areas of study (114 majors) as well as 222 allied, non-FARNRE majors were determined using preliminary 2017-2018 degrees conferred data from the Integrated Postsecondary Education Data System (IPEDS) surveys conducted by the National Center for Education Statistics (NCES) of the U.S. Department of Education. Utilizing 336 total NCES Classification of Instructional Programs (CIPS), graduates from degree programs in FARNRE will meet approx. 61 % (36,100) of the new graduate pool joining the FARNRE workforce, with the balance of graduates (39 %) entering the FARNRE job pool coming from allied, non-FARNRE majors (Fernandez et al., 2020).

Curricular and co-curricular development of professional and interpersonal success skills is vitally important. Employers seeking to hire college graduates increasingly no longer perceive a college degree as the panacea required for meeting today's workforce needs. According to Carlson (2023) many employers believe new college graduates lack some of the desired professional, personal, and success skills expected of a graduate entering the workforce. Two recent workshops and proceedings published by the National Research Council (2009) and the National Academies of Sciences, Engineering, and Medicine (2021) focused on the importance of revitalizing and repositioning curricula and co-curricular opportunities for our students in agricultural and related universities.

In a landmark study, Crawford and Fink (2020) studied critical perceptions, practices and perceived gaps in work and professional related skills in students pursuing degrees from FARNRE institutions. They collected survey data from 31 participating agricultural-related postsecondary institutions across the country, collecting a total of 11,428 survey responses students, faculty, alums, and employers. They found the most important skills identified were foundational skills – listen effectively, communicate accurately and concisely, and identify and analyze problems – while the top skills with the largest preparedness gap were advanced skills – understanding roles and having realistic career expectations, constructively dealing with conflict, and accepting critique and direction. In addition, employers and faculty alike identified certain co-curricular practices and activities deemed important in students developing their employability skills, including holding a job (work), participating in major-related internship and student organizations, participating in research, and serving as a volunteer, to list a few.

## Section 2: An industry overview of needed competencies

Christine Alvarado

Employee skill gaps exist among graduates and the need of employers in the agricultural sector. These gaps seem to be increasing with recent graduates and can create some negative ramifications for the poultry industry. Ramifications for the employee includes such things as limited lack of engagement, high turnover, and employee burnout. In addition, these ramifications can create higher costs for employers due to lack of productivity and higher cost of training employees without longevity of employment. Skill gap ramifications and ways to reduce negative consequences of these skill gaps are important considerations when hiring students for diverse careers in the poultry industry.

A typical hiring process for employees begins with an interview for the skill level required. Once an appropriate candidate for the job skills is found, the employee begins onboarding and learns the day-to-day activities of the job as well as the culture of the workplace. However, this process is only successful if it includes onboarding managers and supervisors with high technical skills, excellent communication skills, excellent motivation, self-driven attitude, confidence, and ability to identify personal success. Unfortunately, recent studies have noted that managers only spend 9 % of their time developing and coaching their direct reports (Baker, 2019). Some suggest this lack of coaching is due to lack of confidence of the managers themselves. Baker (2019) reported that 45 % of managers lack confidence to help employees develop the skills they need today. Other recent research supports the concept of a digital transformation occurring in today's workforce with 70 % of employees lacking mastered skills needed for employment roles (Baker, 2019). Whether it is the managers or the new employees, skills are lacking.

How do employers address these skill gaps? Research by the Brandon Hall group indicates that new hires need a plan of action which starts with proper onboarding (Forry, 2024). They reported that proper onboarding can improve employee retention by 82 % (Forry, 2024). This proper onboarding should include technical soft skills, including one of the most important, communication.

People's needs have changed, and leaders must evolve to support people (employees) better. No longer is getting a paycheck the significant driver for most people. A Mercer report indicated that millennials make up the largest workforce share while Gen Zs are projected to be 25 % of the workforce by 2025. These changes in generational workforces can create significant gaps in what skills are needed for employee success. Understanding these generational gaps and developing an action plan to bridge these gaps is what can lead companies to employee retention and success.

The World Economic Forum (2023) indicated that competencies of a leader are shifting from a current view of things such as analytical thinking, hazard identification, problem solving, and root cause analysis to more human type skills such as self-awareness, compassion, inclusiveness, resilience and curiosity. It is not that current competencies such as analytical thinking are unimportant; however, to have a bigger impact and ensure technical expertise is effectively used, different skills are needed. In addition, the focus on tech and AI means human skills such as those mentioned are even more critical as more interactions take place via technology. On-the-job training is often lacking in these areas which brings a greater need to train these skills in the workplace.

A 2023 Gallup poll indicated that engaged employees, who are involved and enthusiastic about work, make up about 34 percent of the workforce, and actively disengaged employees, who are miserable at work, make up about 16 percent of the workforce. That leaves about 50 percent of employees in the middle who are "meh" about work—they neither love it nor hate it and are probably looking for new jobs. Another report (Mercer, 2019) reported that one in three employees are considering leaving their jobs.

For these reasons, there is a need for a defined plan of action (onboarding plan) for new employees to develop both technical and just as important human or soft skills to ensure success. By actively engaging employees in these plans of action, there is an improved outcome for professional and personal growth (Alchemy). Studies have shown that companies who dedicate their training to specific goals see an increase in employee productivity (15 %), decrease in employee turnover (26 %), less employee absenteeism (20 %), and increase in share prices (65 %).

One of the easiest ways to develop a strong engaged culture as a manager is to ask questions, allow time for reflection and processing of ideas and thoughts, and create a culture of learning, education, work life harmony, profitability and success.

Questions for leaders:

• Does my team have a manageable workload?
• Does my team have time away from work to unplug and recharge fully?
• Does my team find meaning in their work?
• How can I invite my team to lead differently during the “culture” journey?
• What skills are lacking and what targeted training can be conducted to ensure engagement and learning and more human interaction skills?
• How can communication be improved?

Students are no longer learning the necessary soft skills needed for success prior to entering the workforce. There are many reasons for this including the digital age transformations we are witnessing over generations. However, employers’ needs for these skills has not diminished. Therefore, to bridge the gap, a targeted onboarding plan that allows for technical learning and as well as soft skill development is critical to success in our industry.

## Section 3: Strategies for enhancing student preparedness for the poultry industry

Nada Tamim

In today's rapidly evolving job market, ensuring that Poultry Science graduates possess the essential skills required by employers is more critical than ever. The National Association of Colleges and Employers (NACE, 2024) has identified 8 key competencies that are vital for career readiness: teamwork, communication, critical thinking, leadership, professionalism, technology use, equity and inclusion, and career and self-development. However, recent reports indicate that graduates often fall short in these areas, highlighting a significant disconnect between how students and employers assess proficiency in these competencies. For instance, while 79.4 % of students believe they are "very or extremely" proficient in communication, only 55.2 % of employers agree (NACE, 2024).

To bridge the gap between academic preparation and industry expectations, undergraduate programs must adopt a comprehensive approach that aligns the curriculum, co-curricular activities, and career development resources with the key skills employers require. This paper outlines a comprehensive approach with effective strategies that can be implemented during undergraduate education to enhance student preparedness for careers in the poultry industry, focusing on the development of key competencies identified by NACE.

1. **Student Ownership**
  Encouraging students to take ownership of their career readiness is crucial. This process begins with helping them understand the importance of career readiness competencies, employer expectations, and how to develop these skills throughout their undergraduate education. When students recognize the value of these competencies early in their college journey, they are more motivated to engage in experiences and seek opportunities that foster their development. Ultimately, students who take charge of their career readiness are better positioned to meet industry demands and achieve long-term professional success.
2. **Scaffolded Integration in the Curriculum**
  Embedding competency development into the curriculum from the first semester provides students with continuous opportunities to practice and reflect on these skills. This involves designing courses and assignments that progressively build on these skills. For example, group projects in introductory courses can evolve into more complex projects in advanced courses, offering a scaffolded approach to competency development. Through active participation in discussions, group projects, and hands-on activities, students develop critical thinking, problem-solving, and communication skills essential in the workplace. Integrating an academic and career development course into the first semester can significantly benefit students by laying a strong foundation for career readiness to build upon.
3. **Facilitated Engagement**
  Competency development extends beyond the classroom. Facilitating student engagement through internships, research projects, and extracurricular activities allows students to apply their knowledge in real-world settings. These experiences not only enhance technical skills but also provide opportunities to develop needed competencies such as communication, leadership, and teamwork. Programs can support this by requiring internships and building strong partnerships with industry stakeholders, offering networking events, and encouraging student participation in professional activities and reflect on their development.
4. **Advising and Mentoring**
  Effective advising and mentoring are critical components of student development, playing a vital role in complementing the guidance of professional advisors. While professional advisors offer essential guidance on course selection and degree requirements, faculty mentors provide deeper insights into academic disciplines, research opportunities, career pathways, and networking. A dual approach that combines the structured support of professional advisors with the personalized guidance of faculty mentors ensures that students receive both the academic guidance needed for success and the tailored mentorship essential for personal and professional development. Furthermore, incorporating peer mentors during the first semester offers students valuable support and guidance as they transition into college life. Peer mentors can help new students navigate academic challenges, build confidence, and develop essential skills, fostering a sense of belonging and encouraging early engagement in their educational journey.
5. **Continuous, Meaningful Feedback and Assessment**
  Regular feedback and assessment are crucial for continuous improvement, helping students recognize their strengths and areas for growth. Meaningful assessments offer constructive feedback, guiding students in identifying areas for improvement and tracking their progress over time. This can be achieved through self-assessments, peer evaluations, and instructor feedback. Additionally, incorporating reflective practices, such as journals or portfolios, enables students to reflect on their career readiness and gain a deeper understanding of their personal and academic development.

Implementing this comprehensive approach prepares Poultry Science graduates to be both academically proficient and equipped with the key competencies needed to succeed in the ever-evolving Poultry Industry.

## Section 4: Integrating generative AI into higher education: Enhancing learning and addressing challenges

Drew Benson

The increasing inevitability of the use of Generative AI (GAI) in the workforce necessitates that educators actively incorporate these technologies into their courses. AI proficiency is rapidly becoming as crucial as basic computer literacy, enabling students to harness these tools effectively in a wide range of fields.

In the context of the poultry industry, where technological advancements continuously reshape practices and operations, training students to be proficient users of GAI is not just beneficial—it is now essential. The ability to harness the power of AI to increase productivity, optimize processes, and solve complex problems will position graduates as valuable assets within the industry. As technology becomes increasingly sophisticated, the demand for individuals who can effectively integrate and leverage AI into their work tasks will only grow.

Awareness of GAI's strengths and weaknesses will equip students with the skills necessary to tackle the ongoing challenges in the poultry industry. Graduates will be better equipped to innovate, improve efficiency, and contribute to data-driven decision-making processes. Moreover, this deep understanding will enable them to approach AI with a critical mindset, ensuring that they use these tools ethically and responsibly, minimizing potential risks associated with over-reliance or misuse.

The poultry industry, like many others, is poised to benefit significantly from AI-driven advancements. Evidence already exist that demonstrates the impact of using GAI in many occupations. For example, consultants using AI finished 12.2 % more tasks on average, completed task about 25 % more quickly, and produced 40 % higher quality results than those who did not use generative AI (Dell'Acqua et al., 2023). Whether in generating strategic recommendations for market growth, optimizing operations, enhancing biosecurity measures, or improving animal welfare through predictive analytics, the potential applications of AI by our graduates in the poultry industry are vast. As future leaders and innovators, our students must be prepared not just to use AI, but to drive its development in ways that address both current and emerging challenges within the poultry industry.

### Understanding GAI and Its applications in academia

Generative AI refers to a type of artificial intelligence that creates new content based on learned patterns from existing data. A specific example of this is Large Language Models (LLMs) like ChatGPT, which generate human-like text by predicting what comes next in a sequence. These tools have seen rapid adoption, with ChatGPT reaching 1 million users within five days of its launch. A significant percentage of these users are Millennials or Gen Z, with considerable usage among high school students for academic purposes.

In higher education, including our own classrooms, GAI, specifically LLMs, has become a ubiquitous tool. Faculty and students alike are leveraging this technology in various capacities. Faculty use LLM tools to generate lecture content and exam questions, and assist with writing tasks such as reference letters. The use of LLMs has also enabled faculty to easily develop case studies, structured debates, and various active learning activities. Students employ LLMs to create practice quizzes, generate digital flashcards, develop mnemonics, and summarize articles. Furthermore, students are integrating GAI into their academic work to generate ideas, draft reports, and explore complex topics. While concerns about cheating headline the emergence of LLMs in education, these fears can be mitigated by how we assess in higher education. By embracing the technology in our teaching practices and clearly defining when and where LLMs can be used, we can discourage academic dishonesty and raise the bar for student assessments. This approach provides valuable guidance on how to effectively leverage LLMs, ultimately empowering students to use these tools responsibly. Rather than viewing LLMs as a threat, instructors should see them as an opportunity to enhance learning, preparing students to thrive in a future where such technologies are integral to the workplace.

### Challenges and ethical considerations

Despite its benefits, GAI is not without its flaws. Issues such as hallucinating facts, reasoning errors, and the difficulty in tracing the sources of AI-generated information are significant concerns. Additionally, there are inherent biases in GAI outputs, reflecting the biases present in the training data. Anecdotally, these biases and misinformation are obvious when providing general prompts to LLMs regarding the poultry industry. However, these challenges present an excellent training environment for our students. The limitations require students to be trained to critique, verify, and modify LLM outputs, thereby honing their critical thinking skills. Imprinting this critical analysis of LLM outputs will allow our students to fully leverage the technology while maintaining the crucial role of the "human in the loop" (Mollick, 2024). For example, a recent study demonstrated that such critical analysis is essential for improving work performance (Rathnayake & Gunawardana, 2023). By embedding these skills in our students, we are preparing them not only to use LLMs effectively but also to thrive in the future of work, where the ability to interact skillfully with AI will be a key differentiator.

### Adapting assessments for GAI

In the age of LLMs and with the concerns over academic dishonesty, instructors must rethink traditional academic assessments and consider strategies that adapt to the evolving educational landscape. One approach is to raise the bar of expectations for out-of-class work, designing assignments that demand deeper engagement and more complex tasks, with the understanding that students will likely use LLMs to assist them (Grimes et al., 2023; Idialu, 2024). Alternatively, or additionally, instructors can increasingly flip the classroom, where content delivery happens outside of class, and the application and assessment of content knowledge and critical thinking occur within the classroom (Hernandez et al. 2023; Li & Li, 2024). This shift aligns with the growing push for active learning, a method long recognized for its benefits (Dzaiy & Abdullah, 2024). The advent of LLMs almost necessitates the adoption of active learning in higher education, as it not only reduces the likelihood of cheating but also promotes more authentic assessments that genuinely measure student understanding. Moreover, active learning environments offer the added advantage of developing soft skills—such as communication, collaboration, and problem-solving—that are often underdeveloped yet crucial for our students’ future success.

### Conclusion

The integration of GAI into educational settings presents both opportunities and challenges. While GAI can significantly enhance teaching pedagogy and learning experiences, it also necessitates careful consideration of ethical issues and the adaptation of assessment methods. The presentation concluded by encouraging educators to explore and integrate GAI in their courses, emphasizing its potential to equip students with future-proof skills.

## Symposium conclusion

Workforce development in global industries, such as poultry, is a need that will continue to grow. Each of the sections presented in this paper present a unique aspect of how both academia and industry can continue to partner to meet this need and support a workforce with the skills needed to work with diverse others, both domestic and abroad. Moving forward, we must continue to explore best practices for promoting essential skill development in both our undergraduate programming and in the industry's work culture. This will result in an increased workforce of career-ready students and successful employees.

## Declaration of competing interest

All authors on the manuscript, “Preparing students for diverse careers in the poultry industry”, (Elizabeth Karcher, J. Marcos Fernandez, Christine Alvarado, Nada Tamim, and Drew Benson) have no conflicts of interest to declare for this manuscript.
